# Beyond the Protocol: The Vital Role of Patient Guidance in Modern Gastroenterology and Hepatology

**DOI:** 10.1111/jgh.70402

**Published:** 2026-04-27

**Authors:** Han‐Mo Chiu

**Affiliations:** ^1^ Editor‐in‐Chief, Journal of Gastroenterology and Hepatology

The practice of gastroenterology and hepatology is increasingly governed by a framework of rigorous evidence. As clinicians, we look to clinical practice guidelines and consensus statements as our North Star, providing the essential guardrails for standardized, high‐quality care [1]. However, as the complexity of chronic disease management grows, so too does the realization that a “map” of the clinical landscape is not the same as a “guide” for the journey.

In this issue of the *Journal of Gastroenterology and Hepatology (JGH)*, we are proud to feature the article titled “Chronic Hepatitis B Infection: Patient Guidance” [2]. Although this work adheres to the highest standards of scientific evidence, it occupies a distinct and vital niche in our literature—one that is fundamentally different from the consensus statements or clinical guidelines we typically publish.

## 1 A Distinct Role in the Global Literature

Whereas the concept of patient‐centered information is not new, the “Patient Guidance” format in JGH represents a significant evolution. In broader medical literature, we see various formats attempting to bridge the gap between doctor and patient. The JAMA “Patient Page” provides excellent simplified fact sheets for immediate clinical handouts, focusing on basic definitions and treatments [3]. Similarly, the BMJ has pioneered “Patient Perspectives,” often co‐produced with patients to highlight the lived experience of illness [4]. Furthermore, organizations like the European Association for the Study of the Liver (EASL) have begun producing “Lay Summaries” of their technical guidelines to improve accessibility [5].

However, JGH is leading a shift toward what I term “Scholarly Patient Guidance.” Unlike a simplified handout, the guidance published here is a stand‐alone, peer‐reviewed clinical tool. It serves as a “Third Pillar” of medical literature, standing alongside clinical guidelines and consensus statements to ensure that high‐level science actually reaches the individual it is intended to help.

## 2 Practicality Over Prescriptive Power

Clinical guidelines are designed to answer the question: “What is the standard of care?” They are necessarily focused on the strength of evidence and therapeutic thresholds. In contrast, Patient Guidance asks: “How do we manage this condition in the real world?” For the clinician, these articles serve as a bridge. Whereas a guideline tells us when to initiate therapy, this guidance document helps us navigate the nuances of patient counseling—addressing lifestyle habits, psychological burden, and the practicalities of long‐term surveillance that often fall through the cracks of a busy clinic schedule.

## 3 Empowering the Patient: The Impact of Translation

The shift toward value‐based care requires patient empowerment. Chronic hepatitis B (CHB) remains a leading cause of cirrhosis and hepatocellular carcinoma globally, yet its asymptomatic nature creates a significant challenge for adherence.

Notably, the authors of this guidance have gone beyond traditional publication by translating this document into Mandarin and providing it via Open Access. This is a commendable effort toward health equity and literacy. In the Asia‐Pacific region, where the burden of CHB is disproportionately high, removing language barriers is not just a courtesy—it is a clinical necessity. By making this resource freely available in the native language of millions of patients, the authors have provided a tool that can be used directly in the exam room to transform a patient from a passive recipient of care into an active, informed partner.

## 4 Conclusion: A Call for Translatable Science

At the *Journal of Gastroenterology and Hepatology*, our mission is to advance the field with a focus on the needs of our region. We hope this article serves as a template for future submissions. We encourage researchers to continue developing documents that do not just speak *to* experts, but speak *for* the patient. Elevating patient guidance—and ensuring its accessibility through translation—is a significant step toward truly holistic practice in hepatology.

## Funding

The author has nothing to report.

## Data Availability

Data sharing not applicable to this article as no datasets were generated or analyzed during the current study.
